# R0 resection following chemo (radio)therapy improves survival of primary inoperable pancreatic cancer patients. Interim results of the German randomized CONKO-007± trial

**DOI:** 10.1007/s00066-020-01680-2

**Published:** 2020-09-10

**Authors:** R. Fietkau, R. Grützmann, U. A. Wittel, R. S. Croner, L. Jacobasch, U. P. Neumann, A. Reinacher-Schick, D. Imhoff, S. Boeck, L. Keilholz, H. Oettle, W. M. Hohenberger, H. Golcher, W. O. Bechstein, W. Uhl, A. Pirkl, W. Adler, S. Semrau, S. Rutzner, M. Ghadimi, D. Lubgan

**Affiliations:** 1 Department of Radiation Oncology, Friedrich-Alexander-Universität Erlangen-Nürnberg (FAU), Erlangen, Germany; 2 Department of Surgery, Friedrich-Alexander-Universität Erlangen-Nürnberg (FAU), Erlangen, Germany; 3 Department for General- and Visceral Surgery, Medical Center and Faculty of Medicine, University of Freiburg, Freiburg, Germany; 4 Department of Surgery, University Hospital Magdeburg, Magdeburg, Germany; 5 Private practice, Hematology/Oncology, Dresden, Germany; 6 Department of Surgery, University Hospital RWTH Aachen, Aachen, Germany; 7 Department for Hematology, Oncology and Palliative Care, St Josef-Hospital, Ruhr-University Bochum, Bochum, Germany; 8 Department of Radiation Oncology, Universitätsklinikum Frankfurt, Frankfurt, Germany; 9 Department of Medical Oncology and Comprehensive Cancer Centre, Klinikum Grosshadern, Ludwig-Maximilians-University of Munich, Munich, Germany; 10 Department of Radiotherapy, Clinical Center Bayreuth, Bayreuth, Germany; 11 Outpatient Department Hematology/Oncology, Friedrichshafen, Germany; 12 Department of General and Visceral Surgery, Frankfurt University Hospital and Clinics, Frankfurt, Germany; 13 Department of Surgery, St. Josef Hospital, Ruhr-University Bochum, Bochum, Germany; 14 Medical Centre for Information and Communication Technology, Friedrich-Alexander-Universität Erlangen-Nürnberg (FAU), Erlangen, Germany; 15 Department of Medical Informatics, Biometry and Epidemiology, University of Erlangen-Nürnberg, Waldstraße 6, 91054 Erlangen, Germany; 16 Department of General, Visceral and Pediatric Surgery, Medical Center, Georg-August-University Göttingen, Göttingen, Germany

**Keywords:** Pancreatic adenocarcinoma, Surgery, Tumor resectability, Neoadjuvant chemoradiotherapy, Prospective randomized multicenter trial

## Abstract

**Purpose:**

Chemotherapy with or without radiotherapy is the standard in patients with initially nonmetastatic unresectable pancreatic cancer. Additional surgery is in discussion. The CONKO-007 multicenter randomized trial examines the value of radiotherapy. Our interim analysis showed a significant effect of surgery, which may be relevant to clinical practice.

**Methods:**

One hundred eighty patients received induction chemotherapy (gemcitabine or FOLFIRINOX). Patients without tumor progression were randomized to either chemotherapy alone or to concurrent chemoradiotherapy. At the end of therapy, a panel of five independent pancreatic surgeons judged the resectability of the tumor.

**Results:**

Following induction chemotherapy, 126/180 patients (70.0%) were randomized to further treatment. Following study treatment, 36/126 patients (28.5%) underwent surgery; (R0: 25/126 [19.8%]; R1/R2/Rx [*n* = 11/126; 6.1%]). Disease-free survival (DFS) and overall survival (OS) were significantly better for patients with R0 resected tumors (median DFS and OS: 16.6 months and 26.5 months, respectively) than for nonoperated patients (median DFS and OS: 11.9 months and 16.5 months, respectively; *p* = 0.003). In the 25 patients with R0 resected tumors before treatment, only 6/113 (5.3%) of the recommendations of the panel surgeons recommended R0 resectability, compared with 17/48 (35.4%) after treatment (*p* < 0.001).

**Conclusion:**

Tumor resectability of pancreatic cancer staged as unresectable at primary diagnosis should be reassessed after neoadjuvant treatment. The patient should undergo surgery if a resectability is reached, as this significantly improves their prognosis.

## Introduction

Pancreatic cancer is currently the fourth leading cause of cancer-related death worldwide [1]. Palliative chemotherapy remains the only treatment option for the 50% of patients with distant metastases at first diagnosis. According to the National Comprehensive Cancer Network (NCCN) guidelines, pancreatic cancer without distant metastasis is divided into three categories: resectable, borderline resectable, and locally advanced unresectable. Primary surgery is recommended for the 10%–15% of patients who initially have curatively resectable tumors [2]. Patients with borderline resectable pancreatic cancer should receive neoadjuvant treatment followed by surgery, and those with locally advanced unresectable pancreatic cancer should receive palliative treatment [3]. It remains unclear whether patients with initially unresectable pancreatic cancer can achieve resectable status and, if so, whether surgery provides them any additional benefit [4–6]. There are only some case series and small phase II trials supporting surgery in primarily inoperable patients. However, findings from larger prospective studies investigating tumor resectability both before and after treatment are still lacking [7].

The CONKO-007 study was designed to examine the value of radiotherapy in patients with nonmetastatic, unresectable pancreatic cancer [6, 8, 9]; radiotherapy has also been tested in other tumor entities [10, 11]. The protocol stipulates that the resectability of each patient shall be assessed by a panel of five experienced pancreatic surgeons before inclusion in the study to exclude patients with initially resectable pancreatic cancer. Following the neoadjuvant study treatment, the panel surgeons again assess the tumor resectability. The panel surgeons’ recommendations were not binding for the local surgeons, but it was recommended to explore patients with resectable cancer.

Based on the results of a planned interim analysis performed after 180 patients were recruited, independent experts recommended continuation of the study for further examination of the primary outcome (overall survival). Analysis of the secondary outcomes showed that patients who achieved R0 resection following the study treatment had a better prognosis than nonoperated patients. The study steering committee decided to publish these findings because these results may influence the fundamental treatment paradigm for unresectable locally advanced pancreatic cancer. Because these data do not affect the primary outcome of the trial, we consider their publication to be noncritical.

## Material and methods

### Study design and patients

**Fig. 1:**
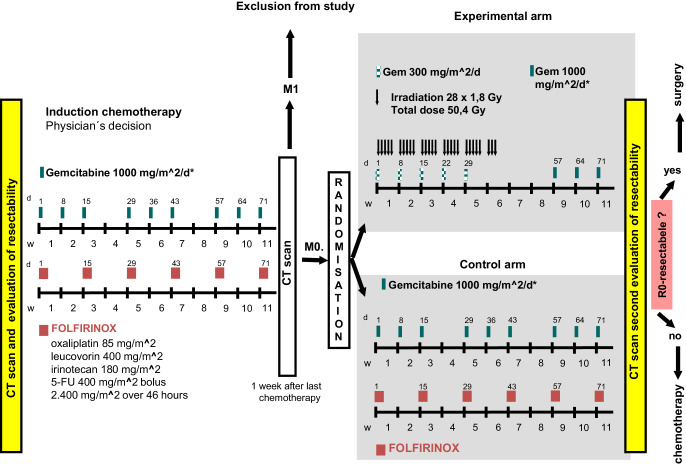
Trial schedule including study inclusion, evaluation for resectability, induction chemotherapy, randomization, therapy after randomization, and subsequent evaluation of resectability at the end of the trial

CONKO-007 is an open-label, multicenter, phase III randomized clinical trial to examine the effectiveness of chemoradiotherapy compared with chemotherapy alone after induction chemotherapy with gemcitabine or FOLFIRINOX (physician’s decision; no randomization) in patients with nonmetastatic, initially locally advanced unresectable pancreatic cancer (Fig. 1).

Eligibility criteria for the trial are age ≥18 years; histologically confirmed, unresectable adenocarcinoma of the pancreas; no evidence of distant metastasis as determined by computed tomography (CT) of the thorax and abdomen; and ECOG performance status ≤2. Patients had to give their written informed consent before participating in the study. The trial was conducted according to ICH GCP guidelines and was approved by the central ethics committee of the University Hospital of Erlangen, Germany (approval no. 322_12AZ) and by the Federal Institute for Drugs and Medical Devices (BfArM, 4038763). Trial registration (EudraCT: 2009-014476-21, NCT01827553) was obtained prior to recruitment.

From 2013 to December 2015, 180 patients were recruited from 52 centers in Germany, more than six patients were enrolled from each of the centers in the following cities: Erlangen, Magdeburg, Göttingen, Freiburg, Dresden, Aachen, Bochum, Frankfurt, Munich, Bayreuth.

After inclusion, each patient’s anonymized diagnostic images (CT or, in exceptional cases, magnetic resonance imaging [MRI]) using a GCP-certified, commercially available clinical trials management system (SecuTrial, interActive Systems, Berlin, Germany [14]) were submitted to five surgeons or an interdisciplinary tumor board, who assessed the resectability status as either “unresectable,” “complete R0 resection possible,” or “R0 resection undetermined” with the aid of a standard questionnaire (Wittel et al., under revision, *BMC Cancer*). The assessment procedure was repeated 4 weeks after the patients completed the study treatment. The panel results were submitted to the centers within 3 work days but were nonbinding. However, the protocol recommendation was to perform surgery in those cases determined to be resectable. Unresectable patients were given the option to continue treatment with chemotherapy outside of the trial.

Induction chemotherapy consisted of either gemcitabine or FOLFIRINOX, depending on the patient’s general health (as determined by the responsible treatment center). Restaging was performed following 6 cycles of FOLFIRINOX or 3 × 3 infusion of gemcitabine. Patients with new distant metastases or those who had received less than two-thirds of the planned chemotherapy were excluded from the study. The remaining patients were randomized to receive either chemotherapy alone with the existing regimen (FOLFIRINOX or gemcitabine) or concurrent chemoradiotherapy with gemcitabine.

Randomization was done with computer-generated block-randomization codes stratified by center, gender, and type of induction chemotherapy (gemcitabine or FOLFIRINOX).

### Chemotherapy (physician’s decision; no randomization)

Gemcitabine 1000 mg/m^2^ was delivered by a 30-min intravenous infusion on days 1, 8, and 15, followed by a repeat cycle on day 29 (days 29, 36, and 43) and day 57 (days 57, 64, and 71). During radiotherapy, patients received gemcitabine 300 mg/m^2^ by a 30-min intravenous infusion on days 1, 8, 15, 22, and 29.

FOLFIRINOX [12] consisted of a 2‑h intravenous infusion of oxaliplatin 85 mg/m^2^ followed by a 2‑h intravenous infusion of leucovorin 400 mg/m^2^ and, after a 30-min break, a 90-min intravenous infusion of irinotecan 180 mg/m^2^. Subsequently, patients received an intravenous bolus infusion of 5‑fluorouracil 400 mg/m^2^ followed by a continuous intravenous infusion of 5‑fluorouracil 2400 mg/m^2^ over 46 h. This cycle was repeated in weeks 3, 5, 7, 9, and 11 (days 15, 29, 43, 57, and 71).

### Radiotherapy

Radiotherapy (intensity-modulated radiation therapy [IMRT] or three-dimensional [3D] radiotherapy techniques) consisted of a total dose of 50.4 Gy in 28 fractions (1.8 Gy/day delivered at a minimum energy of 6 MV). Following induction chemotherapy, the target volume included the primary tumor and suspected lymph nodes with a safety margin of 1 cm. Respiratory motion of the tumor was taken into account in radiation delivery.

### Statistical analysis

Disease-free survival (DFS) was measured from the date of informed consent until one of the following events occurred: distant metastases, local relapse, progress, or death from any cause. For overall survival (OS), death by any cause was defined as event. Patients without any event were censored at the date of their last follow-up. Survival was estimated by Kaplan–Meier analysis. Survival differences were examined with the log-rank test. Differences of R0 resectability before and after treatment, as judged by at least three surgeons per patient, were examined via generalized estimating equations (GEE models). Here, the surgeon’s assessment was used as the independent variable, and the time point (before versus after treatment) was used as the dependent variable. The GEE models account for correlations between assessments of surgeons for the same patients and correlations between patients at different time points. Differences in patient characteristics were examined using Fisher’s exact test and the Kruskal–Wallis test. All tests were two-sided, and the level of significance was set at *p* < 0.05. All statistical analysis was performed using R version 3.4.1[13].

## Results

### Feasibility of induction chemotherapy

Induction chemotherapy consisted of gemcitabine in 43 cases and FOLFIRINOX in 137 cases. Compliance was excellent in both groups: 42/43 patients in the gemcitabine group received a median of 94.8% (mean 86.8 ± 19.5, range 0.0–111.8) of the planned chemotherapy dose, and one patient withdrew before the start of induction chemotherapy. The patients in the FOLFIRIONOX group received a median of 87.0% (mean 80.8 ± 23.6, range 16.4–120.9) of the planned oxaliplatin dose, 87.0% (mean 80.4 ± 24.3, range 8.3–120.9) of the irinotecan dose, and 89.0% (mean 82.2 ± 23.9, range 16.4–120.9) of the leucovorin dose, followed by a median of 85.3% (mean 74.5 ± 30.9, range 0.0–120.9) of the 5‑fluorouracil 400 mg/m^2^ dose delivered by intravenous bolus infusion and 90.6% (mean 82.2 ± 23.8, range 16.4–120.9) of the 5‑fluorouracil 2400 mg/m^2^ dose administered by a continuous intravenous infusion over 46 h.

### Randomization

**Fig. 2:**
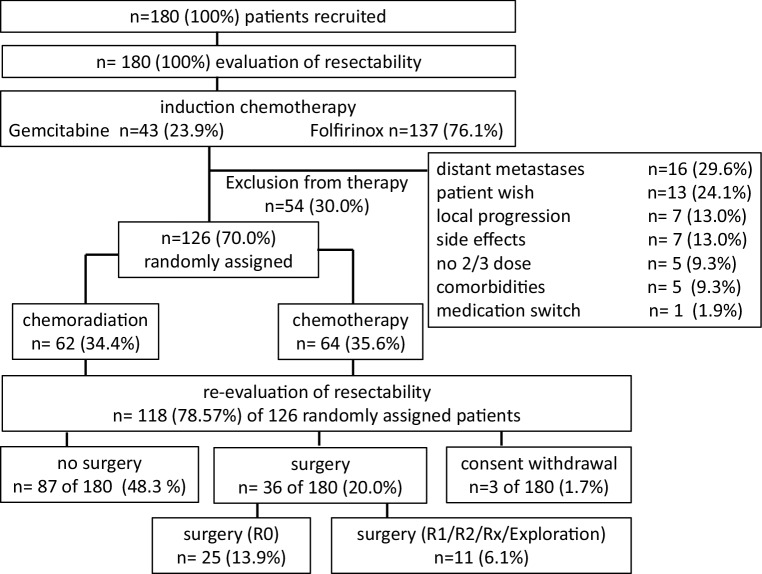
CONSORT diagram

Following induction chemotherapy, 126/180 patients (70.0%) were randomized to further treatment and 54 patients were not (Fig. 2). The main reasons for nonrandomization were distant metastases (29.6%), patient request (24.1%), local progression (13.0%), side effects (13.0%), insufficient dose of induction chemotherapy (9.3%), concomitant disease (9.3%), and treatment switch (1.9%).

### Surgery after completion of study treatment

Following study treatment, 36/180 patients (20.0%) underwent surgery and 87/180 (48.3%) received no surgery. R0 resection was achieved in 25 cases (19.8% of the randomized 126 patients and 13.9% of the overall group of 180 study patients) versus exploration (*n* = 7) or R1/R2/Rx (*n* = 4) resection in 11 cases (6.1%). Patients with R1/R2 resection and explorative surgery were put together because of the low number of patients. Nevertheless, a more detailed analysis—i.e., R1 resection versus R2 resection—must be done in the final analysis, and no conclusions should be drawn from this up to now. The baseline characteristics were well balanced (Table 1).

**Table 1:** Patient characteristics

	All patients	%	R0	%	R1/R2/Rx	%	No surgery	%	*p* value
**Number of patients**	180	100	25	13.9	11	6.1	144	80	N/A
**Age at start of study**	66 (41–79)	N/A	63 (45–73)	N/A	70 (44–79)	N/A	66.5 (41–79)	N/A	0.205 (K-W)
**ECOG at start of study**									
ECOG 0	87	48.3	14	56.0	6	54.5	67	46.5	0.867 (F)
ECOG 1	78	43.3	10	40.0	4	36.4	64	44.5	
ECOG 2	14	7.8	1	4.0	1	9.1	12	8.3	
ECOG missing in the database	1	0.6	0	0	0	0	1	0.7	
**Sex**									
Male	114	63.3	17	68.0	7	63.6	90	62.5	0.909 (F)
Female	66	36.7	8	32.0	4	36.4	54	37.5	
**Tumor stage**									
cT1/cT2	9	5	1	4.0	1	9.1	7	4.9	0.415 (F)
cT3/cT4	134	74.4	21	84.0	10	90.9	103	71.5	
Not available	36	20	3	12.0	0	0	33	22.9	
cT missing in the database	1	0.6	0	0.0	0	0	1	0.7	
**Nodal status:**									
cN0	50	27.8	7	28.0	5	45.4	38	26.4	0.412 (F)
cN+	70	38.9	11	44.0	4	36.4	55	38.2	
Not available	59	32.7	7	28.0	2	18.2	50	34.7	
cN missing in the database	1	0.6	0	0.0	0	0	1	0.7	
**Tumor grading:**									
G1	2	1.1	0	0.0	0	0	2	1.4	0.875 (F)
G2	69	38.3	11	44.0	2	18.2	56	38.9	
G3	50	27.8	6	24.0	4	36.4	40	27.8	
Not available	57	31.7	7	28.0	5	45.4	45	31.2	
G missing in the database	2	1.1	1	4.0	0	0	1	0.7	
**Type of surgery:**									
Pancreatoduodenectomy (Whipple technique)	13	7.2	13	52.0	0	0	–	–	<0.001 (F)
Distal pancreatectomy	7	3.9	5	20.0	2	18.2	–	–	
Total pancreatectomy	6	3.3	5	20.0	1	9.1	–	–	
Core biopsy	0	0	0	0.0	0	0	–	–	
No resection Surgical exploration only	7	3.9	0	0.0	7	63.6	–	–	
Type of surgery unknown	3	1.7	2	8.0	1	9.1	–	–	
No surgery	141	78.3	0	0.0	–	–	–	–	
Consent withdrawn prior to evaluation of resectability at end of study	3	1.7	–	–	–	–	–	–	
*CA19‑9 prior therapy (U/ml)* median (range)	347.2 (0.60–26,081.00)		278.9 (0.60–4613.56)		232.0 (2.0–4743.11)		421.5 (0.70–26,081.0)		0.43 (K-W)
*CEA prior therapy (ng/ml)* median (range)	3.45 (0.50–680.00)		2.9 (0.90–111.10)		2.90 (0.90–104.0)		3.6 (0.50–680.0)		0.216 (K-W)

Data are shown as the median and (range) unless indicated otherwise

*N/A* not applicable, *K‑W* Kruskal–Wallis test, *F* Fisher’s exact test

### Postoperative complications

Five of 36 (13.8%) surgically treated patients developed postoperative complications of grade 3 or worse: bleeding (*n* = 1), pancreatic fistula (*n* = 1), wound healing disturbance (*n* = 1), ileus (*n* = 1), and insufficiency of anastomosis (*n* = 1). Two of 36 (5.5%) patients died from complications: one died on the day after surgery (acute liver failure), and the other died 36 days after surgery (multiple organ failure with sepsis).

### Survival rate as a function of surgical treatment

**Fig. 3:**
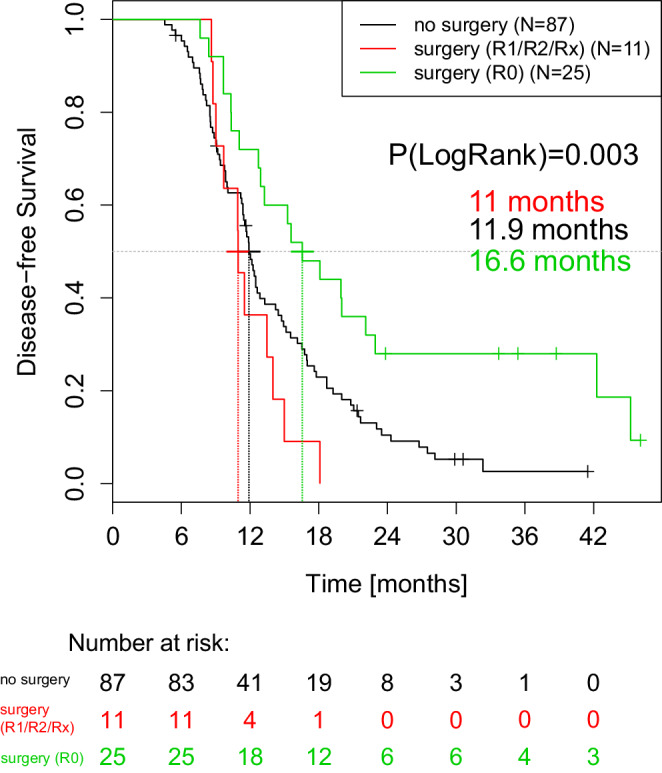
Disease-free survival rates of randomized patients (*n* = 126): R0-resected patients (*green curve*) in relation to R1/R2/Rx-resected patients (*red curve*) and patients without surgery (*black curve*). Median survival rates are given in months

Disease-free survival (DFS) was 11.2 months in the overall population (*n* = 180) compared to 16.6 months in the R0 (*n* = 25) resection group. The prognosis of patients with R0 resection was significantly better (*p* = 0.003) than that of nonoperated patients (DFS 11.9 months) or patients in the exploration or R1/R2/Rx resection group (DFS 11.0 months; Fig. 3). At the 24-month follow-up, the median tumor-free survival rate was 28.0% (95.0% Cl: 14.9–52.5) in the R0 resection group, 10.0% (95.0% Cl: 8.6–0.20.9) in the nonoperated group, and 0.0% in the exploration or R1/R2/Rx resection group (*p* = 0.003).

**Fig. 4:**
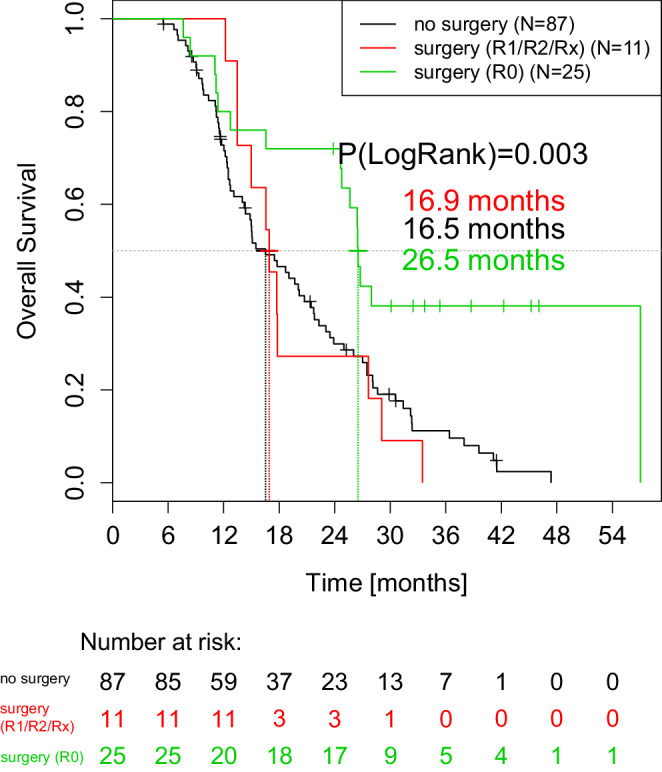
Overall survival rates of randomized patients (*n* = 126): R0-resected patients (*green curve*) in relation to R1/R2/Rx-resected patients (*red curve*) and patients without surgery (black curve). Median survival rates are given in months

These data also carry over to the overall survival (OS) rate. Median OS was 15.0 months in the overall population (*n* = 180) compared to 26.5 months in the R0 resection group (*n* = 25). Patients with R0-resected tumors had significantly better median OS (26.5 months, *n* = 25) than nonoperated patients (16.5 months, *n* = 87) and patients in the exploration or R1/R2/Rx resection group (16.9 months, *n* = 11; Fig. 4). Two-year survival was 72.0% (95% CI: 56.4–91.9) in the R0 resection group compared to 30.0% (95.0% CI: 21.4–41.9) in the nonoperated group and 27% (95.0% Cl: 10.4–71.6, *p* = 0.003) in the exploration or R1/R2/Rx resection group.

### R0 resectability assessments before and after neoadjuvant treatment

All 25 patients with R0 resected tumors were assessed for tumor resectability by at least three surgeons before treatment, and 24/25 were assessed after completing the study. The results of the resectability assessments performed before and after treatment differed significantly, as determined using the GEE model. Each surgeon could submit one resectability decision per case (113 pretreatment resectability decisions for 25 patients and 58 posttreatment resectability decisions for 24 patients). The number of R0 resectable decisions rose from 5.3% (6/113) before treatment to 29.3% (17/58) after treatment (*p* < 0.001, as determined according to the GEE model; Table 2).

**Table 2:** Surgical assessment in the R0 resection group *before* (*n* = 25 patients) and *after* (*n* = 24 patients) the neoadjuvant therapy: tumor resectability was assessed by at least three surgeons. Each surgeon could submit one resectability decision per case

R0 possible	R0 questionable	R0 impossible	No. of cases	R0 possible x no. of cases	R0 questionable x no. of cases	R0 impossible x no. of cases
**Before:**						
2	3	0	1	2	3	0
1	3	1	2	2	6	2
1	2	2	1	1	2	2
1	1	3	1	1	1	3
0	3	0	1	0	3	0
0	2	3	1	0	2	3
0	2	2	3	0	6	6
0	2	1	1	0	2	1
0	1	4	1	0	1	4
0	1	3	2	0	2	6
0	0	5	8	0	0	40
0	0	4	3	0	0	12
*n* (∑113)				**6**	**28**	**79**
**After:**						
3	1	0	1	3	1	0
3	0	0	1	3	0	0
2	1	0	2	4	2	0
2	0	0	1	2	0	0
1	2	0	1	1	2	0
1	1	1	2	2	2	2
1	1	0	1	1	1	0
1	0	0	1	1	0	0
0	3	0	1	0	3	0
0	2	1	1	0	2	1
0	2	0	5	0	10	0
0	1	1	3	0	3	3
0	1	0	1	0	1	0
0	0	3	2	0	0	6
0	0	2	1	0	0	2
0	0	0	1	0	0	0
*n* (∑58)				**17**	**27**	**14**

The proportion of borderline resectable (=R0 questionable) decisions increased from 24.8% (28/113 decisions) before treatment to 46.6% (27/58 decisions) after treatment (*p* = 0.032).

In 23/25 patients, at least one surgeon classified the case as unresectable before treatment compared to only 9/24 cases after treatment (“R0 resection not possible”). Accordingly, the number of “R0 resection not possible” decisions fell from 79/113 (69.9%) before treatment to 14/58 (24.1%) after treatment (*p* < 0.001).

Taken together, these findings show that the pretreatment assessment result was “R0 resection not possible” or “borderline resectable” in the overwhelming majority of cases, but after the study treatment, only 12/24 patients were classified as unresectable or borderline resectable by all surgeons. In other words, neoadjuvant treatment led to a fundamental change in the surgeon’s resectability assessment.

## Discussion

Our interim analysis showed the significance of surgery for this type of cancer, which may be relevant to clinical practice. Therefore, the study steering committee decided to publish these interim results. However, the study is ongoing, so it is not possible to publish any conclusions regarding the primary outcome criteria at this time.

CONKO-007 is one of the first trials in which the resectability of pancreatic cancer is assessed before and after study treatments by an independent panel of surgeons, based on pseudonymized diagnostic images. Only in the study of Katz et al. were the radiographic criteria centrally reviewed before therapy [14]. In other studies, resectability assessments were performed only by local surgeons, with the risk that personal experience, local peculiarities, or other selection factors could influence the assessment results. Moreover, resectability assessment criteria vary from one classification system to another, which leads to additional uncertainty. The use of pseudonymized diagnostic images and standardized questionnaires by our panel ensured maximum objectivity of the resectability assessments. The proportion of R0-resectable decisions prior to the neoadjuvant therapy in our study was only 5.3%, and 69.9% confirmed that resection was surely not possible. This shows that the cohort truly consisted of patients with unresectable pancreatic cancer.

Ultimately, 20.0% of all study patients underwent surgery. R0 resection was achieved in 13.9% of all included patients and 19.8% (25/126) of all those who completed the study treatment. These percentages are lower than those published in the literature [15, 16]. After FOLFIRINOX ± chemoradiotherapy, Hosein et al., Nanda et al., Nitsche et al., Sadot et al., and Marthey et al. achieved R0 resection rates of 30.0%–44.0% [17–21]. Suker et al. in a meta-analysis found that the pooled proportion of patients with resection was 25.9%; R0 resection was 78.4% based on the number of patients with surgery [22].

This variance in the proportions of patients with surgery and R0 resection can be attributed to differences in selection criteria between studies. In our trial, all patients were treated and observed prospectively. Consequently, this cohort should give a more realistic picture of the actual treatment reality. Moreover, because surgery was only a recommendation and not an obligation in this study, the rate might have been even higher if all centers had consistently followed the recommendation. However, this cannot be determined in retrospect. Thus, the resection rate achieved in this study should be regarded as a minimum value.

Median DFS and median OS for our patients with R0-resected tumors were 16.6 months and 26.5 months, respectively. These figures are in line with most of the published data in the literature, especially for pancreatic adenocarcinomas classified as unresectable. Median OS from the start of FOLFIRINOX ranged from 10.0 months to 32.7 months across studies by Hosein et al., Nanda et al., Nitsche et al., Sadot et al., and Marthey et al., with a pooled patient-level median OS of 24.2 months (95.0% CI: 21.6–26.8 months) [17–21]. Moreover, 1‑year OS was 80.0% (95.0% CI: 74.7–84.4), and 2‑year OS was 50.2% (95.0% CI: 42.9–57.5) [22].

Immediate surgery plus adjuvant gemcitabine alone [23, 24] or in combination with capecitabine [23] resulted in comparable median OS times of 24.5–25.5 months and 28 months, respectively.

The treatment results achieved in borderline-resectable pancreatic cancer patient populations are much better. Katz et al., Murphy et al., Yoo et al., and Michelaskos et al. reported that borderline-resectable pancreatic cancer patients who underwent treatment with FOLFIRINOX ± chemoradiotherapy achieved an R0 resection rate of 50%–68.0% [14, 25–27]. Yoo et al. [27] and Michelakos et al. [25] also found that patients who underwent surgical resection had significantly better progression-free survival and overall survival than nonoperated patients. The reported median survival was between 37.7 months and not reached.

The study treatments resulted in a fundamental change in tumor resectability assessments, with an increased rate of “R0 resection possible.” However, the panel surgeons found that 9/24 treatment-completers still were not R0-resectable, which surgery later proved to be incorrect. This attests to the difficulty of making a correct preoperative assessment of tumor resectability after neoadjuvant treatment, as confirmed by Ferrone et al. [28] and Wagner et al. [29]. Although both groups reported that neoadjuvant treatment FOLFIRINOX ± chemoradiotherapy led to a significant decrease in tumor size, CT did not predict the resectability (R0 resection). However, our data suggest that patients with complete resection (R0) benefit from surgery. Due to the low number of patients with R1/R2 resection, no conclusion could be drawn concerning whether there is any benefit of surgery for these patients; here perhaps we need to wait for the results of the final analysis.

The observed postoperative mortality rate of 5.5% in our study was comparable to that of 6.5%–11.5% estimated by Krautz et al., Nimptsch et al., and Zimmermann et al. for German inpatients after major pancreatic cancer surgery [30–32]. Czosnyka et al. did not observe increased postoperative toxicity after neoadjuvant treatment with chemotherapy alone, radiation alone, or combined chemotherapy/radiotherapy before pancreatectomy in 3408 patients [33]. Golcher et al. and Dindo et al., in a randomized study of patients with resectable pancreatic cancer, found no increased toxicity after neoadjuvant chemoradiotherapy [34, 35].

## Conclusions

Our data show that even if pancreatic cancer is staged as unresectable at primary diagnosis, tumor resectability should be reassessed after neoadjuvant treatment. If healthy enough, patients with a good probability of R0 resection should undergo surgery, as this significantly improves their prognosis. Therefore, surgery is an option that should be included in the treatment concept for these patients. However, current methods (CT and MRI) for assessing the resectability of pancreatic cancer do not always reliably predict R0 resectability. Therefore, it is necessary to search for new ways to improve R0 resectability assessments.
